# Clinical Predictors of* Pseudomonas aeruginosa* Bacteremia in Emergency Department

**DOI:** 10.1155/2018/7581036

**Published:** 2018-09-24

**Authors:** Yongsoon Choi, Jin Hui Paik, Ji Hye Kim, Seung Baik Han, Areum Durey

**Affiliations:** Department of Emergency Medicine, Inha University School of Medicine, Incheon, Republic of Korea

## Abstract

**Objectives:**

* Pseudomonas aeruginosa* shows higher mortality rate compared to other bacterial infections and is susceptible to a limited number of antimicrobial agents. Considering inadequate empirical treatment of* Pseudomonas* bacteremia has been associated with increased mortality, it is important for emergency physicians to identify infections by* P. aeruginosa*.

**Methods:**

This was a single-center retrospective case-control study to investigate the clinical predictors of patients diagnosed as* Pseudomonas* bacteremia in the emergency department (ED) from June 2012 to December 2016. Patients with blood culture positive for* Escherichia coli* in the same period were chosen as the control group, and type of infection was matched for each patient.

**Results:**

A total of 54 cases with* Pseudomonas* bacteremia and 108 controls with* E. coli* bacteremia were included. In the case group, 76% was community-acquired infection, 44% received inappropriate empirical treatment in the ED, and in-hospital mortality was 30%. Multiple logistic regression showed that respiratory tract infection was an independent risk factor for* Pseudomonas* bacteremia (OR 6.56, 95% CI 1.78-23.06; p = 0.004), whereas underlying diabetes mellitus (OR 0.22, 95% CI 0.07-0.61; p = 0.004) and presentation as urinary tract infection (OR 0.06, 95% CI 0.02-0.18; p < 0.001) were negative clinical predictors.

**Conclusions:**

We suggest that antipseudomonal antibiotics should be considered beyond simple coverage of Gram-negative bacteria in the ED, especially if the patient is likely to have pneumonia. Having diabetes or presenting with urinary tract infection could be clinical factors unfavorable to use of antipseudomonal antibiotics.

## 1. Introduction

*Pseudomonas aeruginosa* infection shows a higher mortality rate compared to other bacterial infections including* Staphylococcus aureus* or other Gram-negative bacteria [1] and often occurs in patients with underlying malignancy and immunosuppression [2]. Al-Hasan et al. reported that the 28-day all-cause mortality of monomicrobial* P. aeruginosa* bacteremia was 25.5% in a population-based incidence study [3]. Unfortunately,* P. aeruginosa* is susceptible to a limited number of antimicrobial agents suggesting its high mortality could be due to not only inherent virulence, but also delay in administration of appropriate empirical antimicrobial therapy.

It has been documented that inappropriate antimicrobial therapy is associated with adverse outcomes, especially in serious infections caused by Gram-negative bacilli such as* P. aeruginosa *[4, 5]. Given that emergency physicians treat patients with infections empirically before culture results are available, prompt recognition of* Pseudomonas* infection and timely initiation of effective antimicrobial therapy are of great importance. Thus, we performed the case-control study to identify the clinical predictors for* Pseudomonas* bacteremia in the emergency department (ED). In order to determine risk factors, we included a control group consisted of patient with* Escherichia coli* bacteremia, which is most frequently isolated Gram-negative bacilli from blood.

## 2. Methods

### 2.1. Study Design

This was a single-center retrospective case-control study to investigate the characteristics and risk factors of patients diagnosed as* Pseudomonas* bacteremia in the ED from June 1, 2012, to December 31, 2016. This study was conducted in a university hospital in Korea which is a tertiary hospital with 60,000 patients according to an annual census of ED visits.

### 2.2. Study Protocol and Population

A 1:2 matched case-control study was performed by a blinded observer who was unaware of the clinical outcomes. The records of all positive blood cultures collected at the ED over the 4 years were reviewed from the computer database. Patients were included in the study if their blood cultures were drawn in the ED and the culture results were positive for* P. aeruginosa* or* E. coli. *Patients with polymicrobial bacteremia were excluded, and only the first bacteremia episodes for each patient were included in the analysis. This review identified 54 adult patients (age, ≥ 18 years) with clinically significant* Pseudomonas *bacteremia (defined as the case group). Patients with blood culture positive for* E. coli* in the same period were chosen as the control group. Type of infection was matched for each patient.

### 2.3. Variables

The following variables were collected for the case and the control group by reviewing the medical charts: age, sex, comorbidities, predisposing conditions (indwelling urinary catheter, central venous catheter, nasogastric tube, tracheostomy), history of hospitalization or surgery within a year, prior drug exposure including immunosuppressant and chemotherapy within a month, and primary site of infection. Vital signs and results of laboratory tests including neutropenia of enrolled patients were recorded upon ED arrival. Clinical outcome variables such as vasopressor use, intubation, intensive care unit (ICU) admission, and hospital days were also documented.

We investigated antimicrobial susceptibility and multidrug-resistance of the isolated* Pseudomonas *strains and evaluated appropriateness of the empirical therapy in the ED. In-hospital mortality was the main outcome measurement, yet case patients were followed until one year after bacteremia and 1-year mortality was recorded.

### 2.4. Definitions

Clinically significant bacteremia was defined as at least one positive blood culture, together with clinical features compatible with systemic inflammatory response syndrome (SIRS) [6].* Pseudomonas* bacteremia was diagnosed when at least one of the blood sample cultures was positive for* P. aeruginosa*. Source of bacteremia was determined by two independent doctors based on patients' symptoms, physical examination, and radiological findings. Central venous catheter was defined as all dialysis catheters and medication ports, peripherally inserted central catheters. Neutropenia was defined as a neutrophil count lower than 500/mm^3^ upon ED arrival. Shock was defined as either systolic blood pressure < 90 mmHg or a requirement for the use of a vasopressor to maintain blood pressure in the ED.

Case patients were classified as hospital-acquired, healthcare-associated, or community-acquired [7]. Infections were considered to be hospital-acquired if the patients were transferred from another hospital after > 48h hospitalization. Healthcare-associated infections were defined in accordance with the definitions of Friedman et al. [8]: (1) patients received intravenous therapy, wound care, or specialized nursing care at home; (2) patients attended a hemodialysis clinic in the 30 days before the infection; (3) patients resided in a nursing home or long-term care facility. When patients who did not fall into either criteria, they were categorized as having community-acquired bacteremia.

*Appropriateness of Antimicrobial Agents*. The initial empirical antimicrobial therapy was considered “appropriate” if the initial antibiotics included at least one antibiotic that was active in vitro antimicrobial susceptibility testing results and when at least the minimum recommended dose was administered via the intravenous route [9]. Otherwise, initial antimicrobial therapy was considered “inappropriate”.

*Multidrug-Resistance*. Among the automated antibiotic susceptibility test results, "intermediate" and "resistance" were categorized as nonsusceptible. Multidrug-resistance (MDR) was defined as* Pseudomonas* strains resistance to 3 or more of the following eight antipseudomonal agents [10]: *β*-lactam/*β*-lactam inhibitors (piperacillin-tazobactam), cephalosporins (ceftazidime, cefepime), carbapenems (imipenem, meropenem), quinolones (ciprofloxacin, levofloxacin), and aminoglycosides (amikacin).

### 2.5. Microbiology and Antimicrobial Susceptibility

Bloodstream isolates were identified at the species level using the VITEK 2 (bioMérieux Inc., USA). Susceptibility results were interpreted according to Clinical and Laboratory Standards Institute criteria [11].

### 2.6. Statistical Analyses

Data with a normal distribution were expressed as mean ± standard deviation and were analyzed by independent samples* t* test. Data with a skewed distribution were expressed as medians and interquartile ranges and were analyzed by Mann–Whitney* U* test. Categorical variables were compared using *χ*^2^ test or Fisher exact test depending on the sample size. Univariate analysis followed by multivariable logistic regression analysis was performed in this case-control study to identify independent risk factors. Variables with a* p *value of < 0.10 in the univariate analysis were candidates for multivariate analysis using a backward elimination method. A* p* value < 0.05 was considered statistically significant. All statistical analyses were performed using Medcalc for Windows, version 17.6 (MedCalc software, Ostend, Belgium).

## 3. Results

### 3.1. Study Population and Clinical Characteristics

Table 1 shows clinical characteristics of study population. During the study period, a total of 54 patients with* Pseudomonas* bacteremia were identified and included in the analysis as case group. The most common underlying diseases were malignancy (solid tumor and hematologic malignancy, 55%). As shown in Table 2, community-acquired type was most prevalent (76%) among patients with* Pseudomonas* bacteremia, next to hospital-acquired (17%) and healthcare-associated (7%) regarding type of infection. A total of 108 patients with* E. coli* bacteremia matched for type of infection during the period were compared to case patients.

Case patients were more often male (52% versus 28%; p = 0.002) and younger than control patients (66.4 versus 73.5 years; p = 0.021). As for comorbid conditions, solid tumor (46% versus 24%; p = 0.004) and hematological malignancy (9% versus 1%; p = 0.016) were more prevalent in the* Pseudomonas* group. In contrast, diabetes mellitus (20% versus 44%; p = 0.003) was more prevalent in the* E. coli* group. In the case group, history of prior hospitalization and surgery within 1 year was more frequently observed. Moreover, 31% of the case group received chemotherapy within 1 month compared to 4% of the control group (p < 0.0001).

When the sources of bacteremia were evaluated, respiratory tract (39% versus 5%; p < 0.0001), skin and soft tissue (9% versus 0%; p = 0.003), and unknown primary site (7% versus 0%; p = 0.011) were more frequent in the* Pseudomonas* group, yet urinary tract infection (15% versus 78%; p < 0.0001) was more significantly common in* the E. coli* group. Upon ED arrival, case group presented with less higher body temperature and lower level of hemoglobin and HCO3- compared to the control group. In addition, presentation with neutropenia was more prevalent in the* Pseudomonas *group (28% versus 0%; p < 0.0001).

### 3.2. Treatment Outcomes

Table 1 shows that case patients were more frequently intubated in the ED (26% versus 9%; p = 0.005) and ICU care was more common compared to the control patients (44% versus 26%; p = 0.17). The in-hospital mortality was significantly higher in the* Pseudomonas* group than the* E. coli *group (30% versus 8%, p <0.001).

### 3.3. Clinical Predictors for* Pseudomonas* Bacteremia

As shown in Table 3, being male, underlying solid tumor, history of hospitalization, or surgery within a year was associated with* Pseudomonas* bacteremia in the ED by simple logistic regression, yet was not statistically significant in multiple logistic regression analysis. Notably, odds ratio of hematologic malignancy, history of chemotherapy within a month, and presence of neutropenia upon ER visit were 10.91 (95% CI 1.24-95.96; p = 0.031), 11.94 (95% CI 3.77-37.8; p < 0.001), and 41.1 (95% CI 5.25-321.99; p < 0.0001), respectively.

Multiple logistic regression showed that respiratory tract infection was an independent risk factor for* Pseudomonas* bacteremia in the ED (OR 6.56, 95% CI 1.78-23.06; p = 0.004), whereas age (OR 0.96, 95% CI 0.92-0.99; p = 0.029), underlying diabetes mellitus (OR 0.22, 95% CI 0.07-0.61; p = 0.004), and presentation as urinary tract infection (OR 0.06, 95% CI 0.02-0.18; p < 0.001) were negative clinical predictors for* Pseudomonas* bacteremia.

### 3.4. Antimicrobial Susceptibility of Isolated* P. aeruginosa*

Of the 54 strains of* P. aeruginosa* evaluated, the aztreonam showed the lowest susceptibility rate (50%) among antibiotics tested (Table 2). Susceptibility rates of other antibiotics ranged from 80% to 96%. Carbapenems and colistin were effective against 83% and 100% of the isolates. MDR strains (resistant to ≥ 3 antibiotic groups) were identified in 12 isolates (22%).

The choice in empirical antibiotics was made by the emergency physician in charge of the patient. Notably, among the patients with* Pseudomonas* bacteremia, 44% (24 out of 54) received inappropriate antimicrobial therapy in the ED. During one-year follow-up after bacteremia, 33 out of 54 case patients died resulting in 1-year all-cause mortality of 61%.

## 4. Discussion

*P. aeruginosa* causes infections that usually occur late during hospital stay [12]. Affected patients are often hospitalized in an ICU, are immunocompromised, and have multiple invasive devices [13, 14]. Schechner et al. reported that independent predictors of* Pseudomonas* bacteremia were severe immunodeficiency, age > 90 years, antimicrobial therapy within 30 days, and presence of a central venous catheter or urinary device [15]. In contrast to these previous reports focusing on all cases of* Pseudomonas* bacteremia, we limited study population to cases presented to the ED. According to our study, 76% of* Pseudomonas* bacteremia presented to the ED over the 4 years was community-acquired suggesting that possibility of* P. aeruginosa *infection should not be overlooked in the community setting.

Although MDR* Pseudomonas* infections have been increasing since 2000s [16], susceptibility rates of the isolated* P. aeruginosa* in our study ranged from 80% to 100% except for aztreonam with a susceptibility rate of 50%, and 22% of them revealed MDR. Despite this favorable profile of susceptibility, 24 out of 54 case patients (44%) still received inappropriate empirical treatment in the ED, which implies low suspicion could be a matter regarding treatment of* Pseudomonas* bacteremia in the ED rather than MDR* P. aeruginosa.*

In the present study, in-hospital mortality was 30% in line with previous observations [17], yet 1-year mortality was higher [3] probably due to high prevalence of cancer in the study population. In several studies, inadequate empirical treatment of* Pseudomonas* bacteremia has been associated with increased mortality [2, 18]. Lodise et al. who found that delaying appropriate therapy for approximately 2 days significantly increases the risk of mortality in patients with* Pseudomonas* bloodstream infection [19]. However, overuse of antipseudomonal agents due to concern of delaying treatment could increase resistance rates leading to limit future options [20]. Therefore, it is very important for emergency physicians to identify patients who are at risk of developing* Pseudomonas* infection and use appropriate empirical therapy in the ED.

In our study, we presented our recent experiences on* Pseudomonas* bacteremia in the ED, described its clinical characteristics, and tried to identify potential risk factors of* Pseudomonas* bacteremia by comparing patients with* E. coli* bacteremia. We chose* E. coli* bacteremia as the control group in order to narrow clinical scenario into when sepsis caused by Gram-negative bacteria is suspected in the ED. Because antipseudomonal antibiotics already include coverage of common Gram-negative bacteria such as* E. coli*, we expect our results could help emergency physicians select empirical antibiotics properly in the ED, for example, ceftazidime over cefotaxime or piperacillin-tazobactam over ampicillin-sulbactam. Our results showed that respiratory tract infection was an independent risk factor for* Pseudomonas* bacteremia in the ED, which was consistent with the recent findings of Hammer KL et al. [21].

## 5. Limitations

The main limitation of the present study is that it was a single-center retrospective study and the number of case patients with* Pseudomonas* bacteremia was small. Also, we only included clinically significant bacteremia which was defined as positive blood culture with SIRS, and prior antibiotic use was not assessed as a risk factor for* Pseudomonas* bacteremia.

Therefore, additional factors related to* Pseudomonas* bacteremia may not be revealed. In addition, our study did not refer to all patients presenting to the ED with bacteremia, but only patients with documented Gram-negative bacteremia.

## 6. Conclusions

In conclusion,* P. aeruginosa* is an important pathogen in communities as well as in hospitals. The mortality rate of* Pseudomonas* bacteremia was significantly high compared to that of* E. coli* bacteremia in our study; thus higher degree of suspicion in the ED is required to provide appropriate antimicrobial therapy. We suggest that antipseudomonal antibiotics should be considered beyond simple coverage of Gram-negative bacteria in the ED, especially if the patient is likely to have pneumonia. Having diabetes or presenting with urinary tract infection could be clinical factors unfavorable to use of antipseudomonal antibiotics.

## Figures and Tables

**Table 1:** Comparison of clinical characteristics of 54 case patients with *Pseudomonas* bacteremia and of 108 control patients with *E. coli* bacteremia in the ED.

Characteristics	Case (n = 54)	Control (n = 108)	P value
Age	66.4 ± 15.2	73.5 (71.0-76.0)	0.021^*∗*^
Male, no. (%)	28 (52)	30 (28)	0.002^*∗∗*^

Comorbid conditions, no. (%)			
Diabetes mellitus	11 (20)	47 (44)	0.003^*∗∗*^
Cardiovascular disease	10 (19)	24 (22)	0.586
Respiratory disease	1 (2)	7 (7)	0.201
Chronic renal failure	7 (13)	14 (13)	1.0
Liver cirrhosis	3 (6)	5 (5)	1.0
Rheumatologic disease	1 (2)	8 (7)	0.274
Neurodegenerative disease	9 (17)	27 (25)	0.230
Solid tumor	25 (46)	26 (24)	0.004^*∗∗*^
Hematological malignancy	5 (9)	1 (1)	0.016^*∗*^

Predisposing conditions, no. (%)			
Indwelling urinary catheter	3 (6)	10 (9)	0.546
Presence of central venous catheter	4 (7)	3 (3)	0.223
Nasogastric tube in place	2 (4)	2 (2)	0.601
Tracheostomy	1 (2)	2 (2)	1.0

Prior hospitalization within 1 year, no. (%)	40 (74)	52 (48)	0.001^*∗∗*^
Prior surgery within 1 year, no. (%)	13 (24)	10 (9)	0.011^*∗*^
Drug exposure within 1 month, no. (%)			
Immunosuppressant	2 (4)	7 (6)	0.718
Chemotherapy	17 (31)	4 (4)	<0.0001^*∗∗∗*^

Source of bacteremia			
Respiratory tract	21 (39)	5 (5)	<0.0001^*∗∗∗*^
Gastrointestinal tract	17 (31)	23 (21)	0.157
Urinary tract	8 (15)	84 (78)	<0.0001^*∗∗∗*^
Skin and soft tissue	5 (9)	0	0.003^*∗∗*^
Central venous catheter	0	0	
Unknown	4 (7)	0	0.011^*∗*^

Vital signs on presentation			
SBP, mm Hg	117.4 ± 29.3	125.7 ± 24.7	0.061
DBP, mm Hg	70.8 ± 16.3	72.6 ± 15.6	0.514
PR, beats/min	104.4 ± 22.2	100 (94-104)	0.409
RR, breaths/min	19 (18.0-21.3)	20 (18-20)	0.820
Body temperature, °C	37.8 ± 1.2	38.4 (38.1-38.8)	0.025^*∗*^
Saturation, %	97 (95-98)	96 (96-97)	0.436

Laboratory findings on presentation			
Leukocyte count, x10^9^cells/mL	8810 (6729-12623)	10475 (9303-11099)	0.223
Neutropenia	15 (28)	0	<0.0001^*∗∗∗*^
Hemoglobin, g/dL	10.4 ± 2.3	11.6 ± 1.7	<0.001^*∗∗∗*^
Platelet, x10^3^/*μ*l	178.0 (141.9-213.6)	181 (163-197)	0.816
CRP, mg/dL	10.46 (7.87-17.53)	9.64 (7.79-11.5)	0.464
Lactic acid, mmol/L	2.25 (1.43-4.30)	2.50 (2.19-2.90)	0.642

Arterial blood gas pH	7.44 (7.40-7.46)	7.44 (7.43-7.46)	0.310
PCO_2_, mm Hg	28.9 (25.8-31.1)	29.8 (28.5-30.8)	0.252
PO_2_, mm Hg	77.1 (69.8-83.9)	75 (72.3-77.9)	0.338
HCO_3_^−^, mmol/L	18.2 ± 6.2	21 (20.1-21.8)	0.036^*∗*^
SpO_2_, mm Hg	95.7 (95.3-97.0)	95.6 (95.0-96.3)	0.572

Presentation with shock, no. (%)	20 (37)	26 (24)	0.063
Use of vasopressor, no. (%)	20 (37)	25 (23)	0.063
Intubation, no. (%)	14 (26)	10 (9)	0.005^*∗∗*^
ICU care, no. (%)	24 (44)	28 (26)	0.017^*∗*^
Hospital days	9 (3-15)	8 (6-9)	0.980
In-hospital death, no (%)	16 (30)	9 (8)	<0.001^*∗∗∗*^

^*∗*^ p<0.05, ^*∗∗*^ p<0.01 ^*∗∗∗*^ p<0.001: significant change from baseline values.

**Table 2:** Additional characteristics of patients with *Pseudomonas *bacteremia in the ED including antimicrobial susceptibility profile of the isolates.

Characteristics	Case (n = 54)
Infection type	
Community-acquired	76%
Healthcare-associated	7%
Hospital-acquired	17%

Antimicrobial susceptibility	
Aztreonam	50%
Ceftazidime	85%
Cefepime	83%
Piperacillin-Tazobactam	81%
Ciprofloxacin	80%
Amikacin	96%
Imipenem	83%
Meropenem	83%
Colistin	100%

Multidrug-resistance	22%
Use of inappropriate antibiotics	44%
1-year mortality, no (%)	33 (61)

**Table 3:** Logistic regression analysis of clinical predictors of patients for *P. aeruginosa *bacteremia in the ED.

Characteristics	Simple logistic analysis		Multiple logistic analysis	
	OR (95% CI)	P value	OR (95% CI)	P value
Age			0.9 (0.92-0.99)	0.029^*∗*^
Male, no. (%)	2.8 (1.41-5.52)	0.003^*∗∗*^		

Comorbid conditions, no. (%)				
Diabetes mellitus			0.2 (0.07-0.61)	0.004^*∗∗*^
Solid tumor	2.7 (1.35-5.43)	0.004^*∗∗*^		
Hematologic malignancy	10.9 (1.24-95.96)	0.031^*∗*^		

Prior hospitalization within 1 year, no. (%)	3.1 (1.50-6.29)	0.002^*∗∗*^		
Prior surgery within 1 year, no. (%)	3.1 (1.26-7.65)	0.013^*∗*^		

Drug exposure within 1 month, no. (%)				
Chemotherapy	11.9 (3.77-37.80)	<0.001^*∗∗∗*^		

Neutropenia	41.1 (5.25-321.99)	<0.001^*∗∗∗*^		

Primary site of infection				
Respiratory tract			6.5 (1.78-24.06)	0.004^*∗∗*^
Urinary tract			0.06 (0.02-0.18)	<0.001^*∗∗∗*^

^*∗*^p<0.05,  ^*∗∗*^p<0.01  ^*∗∗∗*^p<0.001: significant change from baseline values.

CI = confidence interval; OR = odds ratio.

## Data Availability

The data used to support the findings of this study are available from the corresponding author upon request.
